# Advances in Understanding of Penile Carcinogenesis: The Search for Actionable Targets

**DOI:** 10.3390/ijms18081777

**Published:** 2017-08-16

**Authors:** Juan Chipollini, Sharon Chaing, Mounsif Azizi, Laura C. Kidd, Patricia Kim, Philippe E. Spiess

**Affiliations:** 1 Department of Genitourinary Oncology, Moffitt Cancer Center, Tampa, FL 33612, USA; mounsif.azizi@gmail.com (M.A.); philippe.spiess@moffitt.org (P.E.S.); 2 Morsani College of Medicine, University of South Florida, Tampa, FL 33612, USA; schaing@health.usf.edu; 3 Department of Urology, Temple University, Philadelphia, PA 19140, USA; Laura.Kidd@tuhs.temple.edu; 4 Department of Biological Sciences, University of Notre Dame, South Bend, IN 46556, USA; Patricia.Kim.337@nd.edu

**Keywords:** penile cancer, molecular carcinogenesis, actionable targets

## Abstract

Penile cancer (PeCa) is a rare malignancy with potentially devastating effects. Squamous cell carcinoma is the most common variant with distinct precancerous lesions before development into invasive disease. Involvement of the inguinal lymph nodes is the most important prognostic factor in PeCa, and once disease is present outside the groin, prognosis is poor. Metastatic PeCa is challenging to treat and often requires multidisciplinary approaches in management. Due to its rarity, molecular understanding of the disease continues to be limited with most studies based on small, single center series. Thus far, it appears PeCa has diverse mechanisms of carcinogenesis affecting similar molecular pathways. In this review, we evaluate the current landscape of the molecular carcinogenesis of PeCa and explore ongoing research on potential actionable targets of therapy. The emergence of anti-epidermal growth factor receptor (EGFR) and other immunotherapeutic strategies may improve outcomes for PeCa patients.

## 1. Introduction

Penile cancer (PeCa) is a rare malignancy in Europe and North America [1]. The overall incidence in the United States is approximately 0.69 per 100,000 men and associated with increasing age at diagnosis [2]. Approximately 80% of tumors occur on the glans or prepuce and the most common histology is squamous cell carcinoma (SCC) [3]. The etiology of PeCa is multifactorial with many risk factors identified to date including phimosis, smoking, chronic inflammatory states, number of sexual partners, and human papillomavirus infection [4].

Although rare, PeCa is known for early locoregional and angiolymphatic spread [5]. Thus, there is currently a great need for biomarkers of disease progression and treatment response for this aggressive disease. The most important prognostic factor in the early stages remains the extent of lymph node metastasis at the time of inguinal node dissection with few effective therapies available for those with regional disease present [6]. For those with distant metastatic disease, prognosis remains dismal with most patients succumbing to disease within six months following prior chemotherapy [7]. Although multidisciplinary approaches can be effective in select clinical scenarios, there remains a substantial lack of therapeutic options, particularly targeted therapies, for those with chemotherapy-resistant disease.

In the era of precision medicine, there has been increased interest in the use of targeted therapies, given that the response to standard chemotherapy in advanced PeCa is short-lived; however, the rarity of the disease makes it difficult to perform prospective randomized trials. In addition, the paucity of knowledge of PeCa molecular drivers presents an obstacle in developing novel therapeutic agents. In this study, we review the current understanding of the molecular pathogenesis of penile SCC and explore ongoing research and clinical trials on potential actionable targets of therapy that may help delineate future therapeutic paradigms for advanced PeCa.

## 2. Current Available Therapies in Advanced Penile Cancer

**Figure 1:**
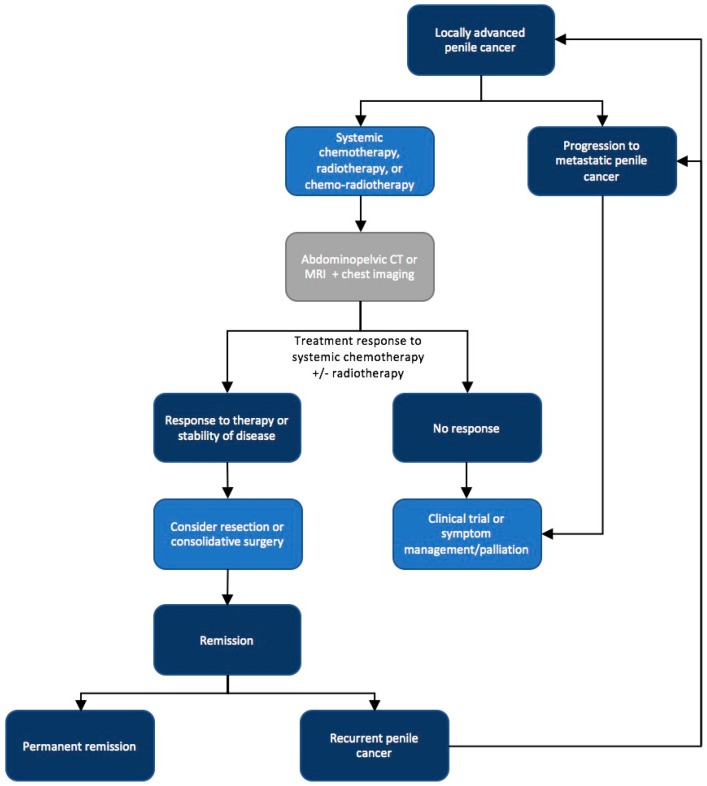
Current treatment paradigm in advanced loco-regional penile cancer. CT: Computed tomography; MRI: Magnetic resonance imaging.

Advanced PeCa is challenging to treat and often requires a multimodal approach involving systemic therapies. Evaluation of the inguinal and pelvic nodes is an essential component during initial evaluation. Even for those presenting with clinically negative groins (cN0), the likelihood of metastatic disease approaches 25% [8]. Chemotherapy as part of combination therapy should be offered to all patients presenting with advanced loco-regional disease [6]. This includes patients with fixed inguinal nodes, palpable nodes ≥4 cm and patients with disease extending into neighboring structures like symphysis pubis and perineum [6,9]. The aim of combination therapy is to allow for surgical consolidation for those fit to undergo surgery (Figure 1).

### 2.1. International Penile Advanced Cancer Trial (InPACT)

Current literature suggests immediate and prophylactic inguinal lymph node dissection (ILND) carries improved survival rather than a delayed or therapeutic ILND [10]. For those with cN0 groins, time and management of the nodes is dependent on primary tumor stage, grade and presence of lymphovascular invasion [11], with early lymphadenectomy having superior outcomes in comparison to awaiting for nodal disease to occur [12]. Current guidelines recommend systemic therapy for those with advanced nodal disease prior to consolidative surgery if responding to treatment, as well as for those exhibiting high-risk pathologic features [13]. However, there has been limited research on the current merits of both neoadjuvant and adjuvant systemic therapies. To effectively examine these issues, the International Rare Cancer Initiative has developed and recently opened the International Penile Advanced Cancer Trial (InPACT) (NCT02305654) to answer this important question. 

InPACT is a multinational, multidisciplinary collaboration which plans on recruiting 400 patients (200 from the UK and 200 from USA and Europe) with locally advanced SCC of the penis over a five year period. The study will help clarify the role of surgery and its integration with multimodal therapies. Patients will be divided into one of the following three treatment arms: 1. ILND with no neodjuvant treatment, 2. neoadjuvant chemotherapy followed by ILND, or 3. neoadjuvant chemoradiotherapy followed by ILND [14]. Additionally, the study will examine whether prophylactic pelvic node dissection (PLND) will improve outcomes in patients at high risk of recurrence. Those at high risk will be identified and divided into a prophylacitc PLND arm versus a surveillance cohort. In conclusion, results from InPACT will undoubtly address some of the current controversies in regional lymph node surgery for PeCa, along with clarifying the optimal timing for systemic therapies in the neoadjuvant or adjuvant settings. 

### 2.2. Current Recommended Systemic Regimens in Metastatic Penile Cancer

**Figure 2:**
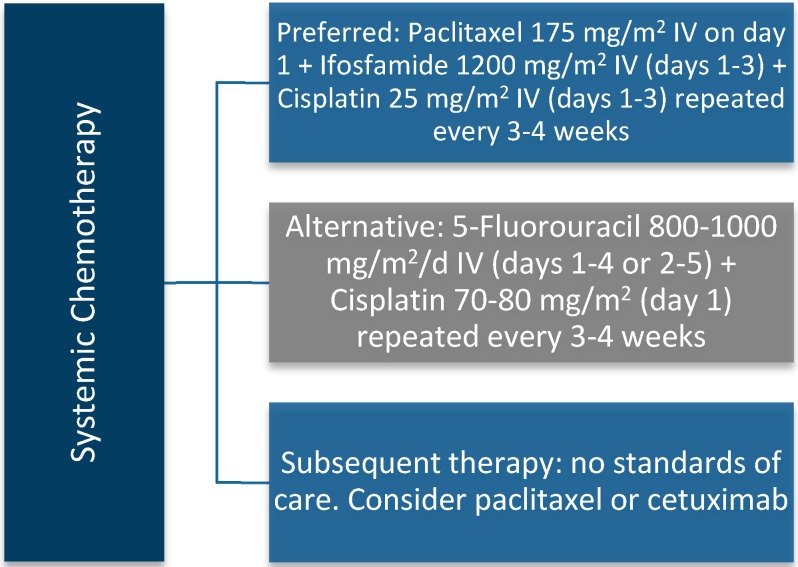
Current regimens in metastatic penile cancer.

Penile cancer even in advanced stages can be responsive to several chemotherapeutic agents. Presently, cisplatin-based regimens (paclitaxel, ifosfamide, and cisplatin [TIP] or fluorouracil [5-FU] and cisplatin) are the most active first-line chemotherapy agents [15,16,17]. TIP is generally well tolerated and is the only regimen evaluated in a prospective study [15]. Cisplatin with 5-FU is also a reasonable alternative although severe neutropenia can be observed in 20% of patients [16]. Vincristine, bleomycin, and methotrexate are also viable options although significant toxicity has been noted [18,19,20]. There are no standard second-line therapy options, although single-agent paclitaxel has shown moderate response in this setting [21]. Unfortunately, due to the low incidence of penile cancer, no large studies have been reported concerning chemotherapy. Figure 2 lists current treatments in advanced penile cancer.

## 3. Mechanisms of Penile Carcinogenesis

Although unique aberrant pathways have been found in various malignancies, molecular mechanisms underlying PeCa carcinogenesis remain poorly understood. At present, PeCa is thought to arise from progression of precursor lesions, and can be subdivided into human papillomavirus (HPV)-dependent and HPV-independent pathways [22]. An overview of these pathways is discussed below. 

### 3.1. Human Papillomavirus (HPV)-Dependent Carcinogenesis

HPV is a DNA virus with more than 100 different known genotypes and has been implicated in the development of penile carcinoma [4,23,24]. Viral infection is usually transient, and it occurs when squamous epithelium maintains virion production which develops into a morphologic low-grade lesion (e.g., condyloma and mild dysplasia). On the other hand, HPV viral-associated precancerous lesions (e.g., penile intraepithelial neoplasia) result from viral genome integration with host genome leading to overexpression of oncogenes that drive cell proliferation and malignant transformation, and become precursors to invasive SCC [24]. 

HPV encodes the *E5*, *E6*, and *E7* oncogenes. However, only *E6* and *E7* oncogenes are necessary for malignant transformation and maintenance of malignant phenotype in host cells [4]. The activation of viral *E5* oncogene is not necessary for malignant transformation; however, it may contribute to carcinogenesis by manipulating viral uptake of host target cells. The *E5* gene product is a transmembrane protein that regulates activation of epidermal growth factor receptor (*EGFR*). *EGFR* upregulation leads to a decrease in E-cadherin expression and associated increase in matrix metalloproteinase (*MMP*)*-9* resulting in decreased cell-to-cell adhesion [25]. The *E6* and *E7* oncogenes contribute to carcinogenesis by disrupting centrosome synthesis required for mitosis. Thus, the development of multipolar mitosis is a hallmark feature of both HPV-mediated premalignant and malignant lesions. Additionally, *E6* and *E7* oncoproteins target the tumor suppressors *p53* and retinoblastoma-1 (*RB1*) genes, respectively [23]. These tumor suppressors are negative regulators of cellular proliferation; thus, inactivation can result in uncontrolled cellular growth.

There are 20 HPV serotypes which are known to infect the genital tract and are generally classified between low-risk (lr) or high-risk (hr) depending on their correlation with cervical malignancy. HPV-16 and HPV-18 have been found in around 31% of penile tumors with HPV-16 being the predominant subtype [26]. In cases of hr-HPV infection, viral *E7* binds to the Rb tumor-suppressor with much higher affinity than low-risk HPV subtypes, such as HPV-6 and HPV-11. One of the major functions of *Rb* is to bind and inhibit transcription factors of the E2F-family which leads to downregulation of products involved in DNA and chromosomal replication [27]. This interference allows for cyclin-dependent kinase inhibitor *p16INK4A* to accumulate in the nucleus and inhibiting G1 cyclin-dependent kinase 4 (*CDKN4*) and *CDKN6*; thus, leading to phosphorylation (inactivation) of the Rb tumor-suppressor protein [28]. Hence, in high-risk HPV-derived tumors, *p16INK4A* overexpression can serve as a surrogate immunohistochemistry (IHC) marker of disease, and could be a target for antigen-specific immunotherapy for men at significant risk of disease recurrence [29]. Although the majority of HPV infections do not develop into pathogenic external lesions, it is clear there is a distinct molecular pathway for HPV-derived downstream molecules and their associated preneoplastic lesions.

### 3.2. HPV-Independent Carcinogenesis

Penile carcinomas that are not a result of HPV infection are thought to be a consequence of precursor lesions in areas of chronic irritation/injury (e.g., lichen sclerosis) that progress into neoplastic lesions. Even though the initial source of these precursor lesions has not been entirely elucidated, inflammation is understood to be fundamental to tumor development in these cases as many penile cancers arise from sites of inflammation [22]. Inflammatory cells produce reactive oxygen/nitrogen species (ROS/RNS) which are involved in the development and progression of several human cancers [30].

A key tumor suppressor gene of ROS/RNS damage is p16. Loss of heterozygosity of the p16 gene has been frequently observed in penile carcinomas; thus, it is possible this pathway plays a crucial role in penile carcinogenesis, specifically in the context of chronic inflammation [31]**.** Other important mediators in inflammation-induced penile carcinogenesis are cyclooxygenase-2 (*COX*-*2*) and prostaglandin E2 (*PGE2*). *COX-2* has been shown to be greatly expressed in PeCa [32]. When *COX-2* is overexpressed, there is an overproduction of prostaglandins and thromboxanes, with *PGE2* specifically playing a critical role in proliferation, angiogenesis, and activation of *EGFR* [33]. Additionally, *PGE2* activates β-catenin-T-cell factor, which supports replicative potential and immortalization; and *PI3K*, which assists in cell migration and invasion [33,34].

Evidence suggests that gene alterations (i.e., p53 alterations, gene promoter methylations) are more frequent in HPV-independent than HPV-mediated tumors [35,36]. As mentioned earlier, HPV-associated carcinomas are characterized by viral oncoproteins that disrupt Rb and p53 pathways. Thus, it seems reasonable that HPV-independent carcinogenesis requires alternative genetic damage that disrupts similar targets. Other mechanisms identified include nonviral disruption of the *p16INK4a/cyclinD/Rb* and *p14ARF/MDM2/p53* pathways [37,38]. Hypermethylation of the *p16INK4a* promotor region (inactivation) has been observed in 15% of hr-HPV negative cases [39]. When considering all the available evidence, it is clear that while HPV-dependent and independent tumors have differences in molecular carcinogenesis, they eventually come to affect similar pathways. While the former uses the activity of viral oncogenes to disrupt tumor suppressor genes, the latter results from genetic alterations that lead to disruption of related tumor suppressing pathways.

## 4. Current Established and Emerging Targets of Therapy

A few recently discovered molecular targets have been reported in the literature with encouraging findings. However, validation continues to be a struggle due to lack of preclinical PeCa systems to validate results. Nevertheless, these initial findings justify clinical trials investigating these novel targets. Table 1 lists currently open clinical trials of systemic molecular targets.

### 4.1. HPV-E6/E7

Targeting the *E6*/*E7* pathways appear to be promising actionable targets of therapy. In the cervical HPV experience, one phase II therapeutic vaccination study using a combination of synthetic plasmids targeting HPV-16 and HPV-18 *E6* and *E7* caused a 40% histopathological regression in women with HPV-16-positive or HPV-18-positive lesions [40]. Another promising advancement involves adoptive T-cell therapy by harvesting patient-specific T cells derived from primary or metastatic foci. These HPV-targeted tumor-infiltrating lymphocytes (TILs) could then be transferred back to donor-patients in order to induce an anti-tumor immune response. In the metastatic cervical cancer setting, one study demonstrated a 33% (3/9) objective response including two long-term complete responses after infusion of HPV-16/18 *E6* and *E7* reactive TILs [41]. Although lymphocyte-depleting chemotherapy was necessary along with a 6-week incubation period, adoptive T-cell therapy appears to be promising in the treatment of HPV-derived cancers and could yield significant insights in treatment approaches in other viral-associated malignancies.

### 4.2. Programmed Death-1 (PD-1)/PD-1 Ligand (PD-L1)

The program death-1 (*PD-1*)/PD-1 ligand (*PD-L1*) axis has been demonstrated to play an important role in tumor immune escape, and immunotherapies targeting this pathway have shown great success in other urologic malignancies [42,43]. Udager et al. first reported the frequent *PD-L1* expression in penile cancer [44]. Approximately 62% (23/37) of tumors were positive for *PD*-*L1* and associated with poor disease specific survival (DSS) (*p* = 0.011) and lymph node metastasis (*p* = 0.024). One large study validated this high proportion of PD-L1 expression in which 48% (96/200) of patients stained positive, and mainly HPV-negative tumors [45]. Although there are limitations and controversies in regards to *PD-L1* immunohistochemical assessment and scoring, these findings indicate *PD-1/PD-L1* as a potential target in PeCa. Trials are being planned using anti-*PD-1* (NCT02837042) or *PD-L1* antibodies (NCT02721732) that may clarify the role of checkpoint inhibition in PeCa. The molecular link between unique genomic features and response to checkpoint inhibitors require further investigation. 

### 4.3. Epidermal Growth Factor Receptor (EGFR)

The human epidermal growth factor receptor (*HER*) family is composed of *EGFR*, *HER2*, *HER3*, and *HER4* transmembrane tyrosine kinase receptors [46]. Reports demonstrate high levels of *EGFR* to be a common feature of penile carcinomas independent of histologic subtype, grade, and HPV status [47,48]; thus suggesting this pathway has a significant role in penile carcinogenesis. The phosphorylated form of *EGFR* is associated with increased risk of recurrence (OR 7.6, *p* = 0.009) and shorter overall survival in N0-1 patients (HR = 9.0, *p* = 0.012) [7]. One published case report demonstrated objective response in a patient treated with the anti-*EGFR* monoclonal antibody panitumumab [49]. A series of 24 patients treated with *EGFR*-targeted drugs cetuximab, erlotinib or gefitinib, alone or in combination, showed a 23.5% partial response with cetuximab having increased antitumor activity for those treated with cisplatin-based chemotherapy [50]. A meta-analysis of 28 patients in total demonstrated 50% response rate with a median progression free survival (PFS) of 3 months (1.5–5.78) [51]. Another review of 65 patients, of which 17 patients were treated with cetuximab-including regimens, showed a trend for improved response (OR = 5.05, *p* = 0.077) when compared to those who received taxane-based regimens alone [52]. These results seem to indicate that although *EGFR* pathway plays an initial role in penile carcinogenesis, those with advanced disease continue to benefit from standard chemotherapy regimens in addition to anti-*EGFR* therapy. A prospective randomized trial evaluating cetuximab and TIP (NCT02014831) was initially set to begin enrollment but was withdrawn due to lack of industry drug supply.

### 4.4. Vascular Endothelial Growth Factor (VEGF)

Antiangiogenic therapy has been effective in the treatment of lung and head and neck SCC, so it can be postulated that antiangiogenic therapy can be effective in PeCa. Zhu et al. evaluated the efficacy of kinase inhibitors (sorafenib and sunitinib) in 6 patients who had previously received at least two chemotherapy regimens. One partial response and 4 stable disease responders were observed [53]. Additionally, cytotoxic agents such as paclitaxel, which have antiangiogenic effects when administered at low doses, along with other antiangiogenics have shown activity in melanoma and urothelial carcinomas. One study combining pazopanib with weekly paclitaxel for patients previously treated with cisplatin-based chemotherapy was terminated due to low recruitment (NCT02279576). It is possible that there are molecularly defined groups who may benefit from *VEGF*-targeted therapy, or, conversely, who may be specifically sensitive to taxanes. More research is needed for this specific second-line treatment.

### 4.5. Human Epidermal Growth Factor Receptor (HER)/Akt/PTEN

Genetic alterations of genes in the *PI3K* pathway have been implicated in various malignancies [54]. Such alterations include loss of the tumor suppressor *PTEN* and amplification of *PIK3CA* and *Akt*. One study found HPV-negative tumors expressed more activated *EGFR* than HPV-positive ones and this expression correlated with activated *Akt*, implicating *EGFR* as an upstream regulator of *Akt* signaling in penile cancer [46]. Conversely, *HER3* expression was significantly more common in HPV-positive cases and positively correlated with cytoplasmic *Akt1* expression. Currently two companion trials targeting the *HER* pathway in advanced penile SCC are underway. One 1^st^-line/neoadjuvant trial using Dacomitinib (NCT0172833), a potent, irreversible kinase inhibitor of human *EGFR/HER1*, *HER2* and *HER4*, has shown anti-tumor activity in N2-3 and M1 patients. Preliminary results have demonstrated a 42.8% (6/14) progression free rate with median PFS of 4.47 months and median overall survival (OS) of 11.9 months. The most common side effects were skin toxicity in 7 patients, diarrhea in 2 and bleeding cutaneous metastasis in one [55]. Updated data on 23 patients found *EGFR* amplification in 4 responders and mutations in *HRAS*, *BRAF*, *PIK3CA*, *PTEN*, and *STK11* in 47% of non-responders with potential associated resistance to *EGFR* inhibitors [56]. Another trial in the salvage setting with Afatinib (NCT02541903) will start accruing. These trials will provide insights into targeting the *HER* pathway with preliminary data thus far linking molecular alterations with clinical response.

## 5. Mutagenesis in Penile Cancer and the Potential for Molecular Targeting

Other than HPV-driven transformation, little is known about the molecular alterations during the development of PeCa. Significant advances in genetic sequencing have allowed discovery of multiple genomic alterations occurring during PeCa progression. For those HPV-positive cases, *MYC* amplification was first reported with integration of HPV DNA sequences [57]. Conversely, HPV-negative tumors have been found to express significantly more phosphorylated *EGFR* than HPV-positive tumors with corresponding increases in *pAkt* expression [46]. Further molecular classification of these tumors along with knowledge of their effect on drug response and tolerability to side effects may allow for individualization of therapeutic regimens and incorporation into clinical guidelines. Recent studies on genetic and molecular pathways implicated in the development of penile cancer are listed in Table 2.

### 5.1. Epigenetic Mechanisms

Epigenetic modifications are potentially reversible alterations in DNA methylation or chromatin that are not associated with changes in DNA sequence [64]. Published studies on PeCa are limited to the evaluation of CpG islands in specific genes [64,65]. Most studies interrogate the CpG status of *CDKN2A* locus which codes the tumor suppressors *p16INK4A* and *p14ARF*. Co-inactivating mutations in *CDKN2A* and *p53* were observed more frequently in lichen sclerosus-derived tumors than in HPV-derived cases (*p* = 0.053) in one study **[66]**. Another study identified *p53* expression along with *p16INK4A* negativity in HPV-negative tumors [28]. Hypermethylation of *CDKN2A* was correlated with negative and weak expression of p16 in one study, with all of the HPV-negative cases having weak or no p16 expression [67]. Lymph node metastasis has been associated with negative p16 expression as well as loss of heterozygosity and promoter hypermethylation of *p16INK4A* [31]. These differences in *p16* expression and methylation differences of *CDKN2A* between HPV-positive and negative tumors support the hypothesis that PeCa develops from distinct molecular pathways with likely inherent differences in therapeutic approaches.

### 5.2. Genetic Profiling and Deep Sequencing

With the arrival of deep targeted sequencing, more studies on genetic alterations have been possible in the last few years. In one of the first molecular studies, next-generation sequencing identified a median of two relevant somatic mutations and one high-level copy-number alteration per sample [48]. Furthermore, advanced stage, lack of *p16* expression, and *MYC* and *CCND1* amplifications were significantly associated with shorter time to progression or survival. Another study using whole exome sequencing of 27 PeCa samples revealed 810 genes containing somatic mutations with a mean somatic mutation rate of 30 per sample [58]. Of note, there was no association between mutational burden and stage, while tumors with high viral load showed lower mutational rates when compared to HPV-negative cases (*p* < 0.05). Another study of 25 patients treated with first-line cisplatin-based chemotherapy evaluated expression of 738 genes using NanoString technology [59]. In univariate analysis, upregulated *MAML2* (*p* = 0.004), *KITLG* (*p* ≤ 0.0001), and *JAK1* (*p* = 0.029) genes were associated with poor OS, and upregulated *FANCA* was associated with better OS (*p* = 0.024). Acquired mutational changes in these genes may help explain mechanisms of resistance to first-line chemotherapy, thus warranting further evaluation as therapeutic targets.

### 5.3. DNA Copy-Number Alterations

Copy number variation, in which a considerable number of base pairs are duplicated or deleted, is a relatively new field in genomics. One group analyzed copy-number aberrations in 24 patients using high density genome wide methylation arrays [60]. Significant amplifications were found including *4p15.2*, *9p22.3*, *19p13.2*, *19p12* and *19q13.2.* Distinct patterns of copy number gains were noted for both the HPV-positive (*1p36.11*, *3q26.2*, *6p22.1*) and lymph node positive samples (*3q26.2* and *11q22.2*). These copy number variations included known oncogenes *MYC* and *FGFR3* as well as tumor suppressors *CDKN2A*, *CCND1*, *RB1*and *p53*. Another study used array comparative genomic hybridization combined with HPV genotyping [61]. Genomic alterations mapped at *3p* and *8p* were related to worse prognostic features including advanced T stage, recurrence, and death from disease. Losses of *3p21.1–p14.3* and gains of *3q25.31–q29* were associated with reduced DSS. Loss of *DLC1* was an independent risk factor for recurrence on multivariate analysis. The study was significant in showing that recurrent copy-number alterations have a prognostic value in PeCa. 

### 5.4. Micro RNA (miRNA) Aberrant Levels

Studies have suggested miRNA, which modulates gene expression at posttranscriptional level, is frequently dysregulated and aberrantly expressed in human cancers [62,68]. One study evaluated the miRNA profile of 10 primary tumors and found notable miRNA aberrations when compared to adjacent normal tissue [62]. Notably, putative target genes of deregulated miRNAs were those involved in cell growth, axonogenesis, and angiogenesis; thus, miRNA alterations appear to play an active role in the transformation of normal cells to malignant lesions. An integrative analysis revealed *MMP1* and *MMP12* may be regulated by *hsa-miR-145-5p*, which was down-expressed in PeCa tissues [69]. Although hsa-miR-145 down-expression did not predict poorer prognosis, its target *MMP1* showed increased expression in patients with lymph node metastasis. A recent study reported loss of *miR-1*, *miR-101*, and *miR-204* associated with lymph node metastasis and unfavorable prognosis [63]. In terms of clinical utility, miRNA signature panels may have a prognostic role in risk stratification of patients at risk for early nodal metastastic disease.

## 6. Conclusions

Metastatic penile cancer has a poor prognosis after treatment with standard chemotherapy agents. The incorporation of molecular panels has the potential to increase available prognostic and therapeutic capabilities. It is clear penile cancer has distinct molecular pathways with diverse genetic and epigenetic changes with potential therapeutic implications. More translational research and collaboration is needed to continue to develop novel diagnostic biomarkers and effective therapeutic strategies for PeCa patients. Thus far, preliminary data on molecular alterations linked to clinical benefits are being reported. A better understanding of the basic biology of penile cancer can help design future prospective trials and offer insights into potential precision medicine approaches for this rare disease.

## Figures and Tables

**Table 1:** Currently open trials of systemic targets in penile cancer.

ClinicalTrials.gov Identifier	Treatment(s)	Outcomes Measured	Estimated Enrollment (Estimated Completion Date)
DART: Dual Anti-CTLA-4 and Anti-PD-1 Blockade in Rare Tumors (NCT 02834013)	Ipilimumab; Nivolumab	Primary: Overall response rate. Secondary: Best response rate, clinical benefit rate, adverse events, OS, PFS	334 (August 2020)
Phase II Study for the Evaluation of Efficacy of Pembrolizumab (MK-3475) in Patients With Rare Tumors (NCT02721732)	Pembrolizumab	Primary: Non-progression rate. Secondary: Overall response rate	250 (August 2019)
A Phase I Trial of T Cell Receptor Gene Therapy Targeting HPV-16 E7 With or Without PD-1 Blockade for HPV-Associated Cancers (NCT02858310)	E7 TCR transduced cells; Pembrolizumab; Aldesleukin; Fludarabine; Cyclophosphamide	Primary: Dose of E7 TCR cells plus aldesleukin with or without pembrolizumab for the treatment of metastatic HPV-16+ cancers	180 * (January 2026)
A Phase 1 Study of Cabozantinib Plus Nivolumab (CaboNivo) Alone or in Combination With Ipilimumab (CaboNivoIpi) in Patients with Advanced/Metastatic Urothelial Carcinoma and Other Genitourinary Tumors (NCT02496208)	Cabozantinib S-malate; Ipilimumab; Nivolumab	Primary: Adverse events, recommended phase II dose. Secondary: Clinical response rate, OS, PFS, PD-L1 and MET expression	135 (December 2017)
Phase II Study of the Pan-HER Inhibitor Dacomitinib (PF-00299804) for Patients With Locally Advanced or Metastatic Squamous Cell Carcinoma of the Penis (NCT01728233)	Dacomitinib	Primary: Overall response rate. Secondary: Safety and tolerability, complete response rate, PFS, OS, quality of life score	37 (February 2018)
Phase II Trial of Pembrolizumab for Advanced Penile Squamous Cell Carcinoma Following Previous Chemotherapy NCT02837042)	Pembrolizumab	Primary: Objective tumor response rate. Secondary: Duration of response, PFS, OS, adverse events	35 (October 2020)
HPV-16/18 E6/E7-Specific T Lymphocytes in Patients With Relapsed HPV-Associated Cancers (NCT02379520)	HPV Specific T Cells; Cyclophosphamide; Fludarabine; Nivolumab	Primary: Dose-limiting toxicity. Secondary: Overall response rate	32 (October 2033)

* Multiple cancers including penile cancer. OS: Overall survival; PFS: Progression free survival; PD-1: Programmed death-1; PD-L1: PD-1 ligand; TCR: T-cell receptor

**Table 2:** Recent significant genomic studies in penile cancer.

References	Year	*N*	Method	Genes or Segments Studied	Applicability
McDaniel et al. [48]	2015	43	Next generation sequencing	*MYC*, *CCND1*, *p16*	Causative
Feber et al. [58]	2015	70	TrueSeq whole-exome sequencing	*TP53*, *FAT1*, *CNS1*	Causative
Necchi et al. [59]	2016	25	Nanostring gene profiling	*MAML2*, *KITLG*, *JAK1*, *FANCA*	Prognostic
Rodney et al. [60]	2016	24	Genome wide methylation arrays	*MYC*, *FGFR3*, *CDKN2A*, *CCND1*, *RB1*, *p53*	Causative
Busso-Lopes et al. [61]	2015	46	Array comparative genomic hybridization, FISH and PCR	*3p*, *8p*, *DLC1*	Prognostic
Zhang et al. [62]	2015	10	Next generation sequencing	*MAPK*, *p53*, *Wnt*, *TGF-β* and *PI3K-Akt*	Prognostic
Hartz et al. [63]	2016	24	TaqMan arrays and PCR	*miR-1*, *miR-101* and *miR-204*	Prognostic

## Abbreviations

COX-2	Cyclooxygenase-2
EGFR	Epidermal growth factor receptor
ILND	Inguinal lymph node dissection
DSS	Disease specific survival
HPV	Human papillomavirus
OS	Overall survival
PD-1	Programmed death-1
PeCa	Penile cancer
PFS	Progression free survival
PGE	Prostanglandin
PLND	Pelvic lymph node dissection
Rb	Retinoblastoma
SCC	Squamous cell carcinoma
